# Cystathionine Detection via Proton Magnetic Resonance Spectroscopy (¹H-MRS) for the Diagnosis of Oligodendroglioma: A Case Report

**DOI:** 10.7759/cureus.93087

**Published:** 2025-09-24

**Authors:** Tomoka Nakashima, Kazufumi Kikuchi, Koji Yamashita, Daichi Momosaka, Masaoki Kusunoki, Daisuke Kuga, Ryusuke Hatae, Yutaka Fujioka, Ryosuke Otsuji, Mikiko Hashisako, Osamu Togao, Koji Yoshimoto, Kousei Ishigami

**Affiliations:** 1 Department of Clinical Radiology, Graduate School of Medical Sciences, Kyushu University, Fukuoka, JPN; 2 Department of Molecular Imaging and Diagnosis, Graduate School of Medical Sciences, Kyushu University, Fukuoka, JPN; 3 Department of Neurosurgery, Graduate School of Medical Sciences, Kyushu University, Fukuoka, JPN; 4 Department of Anatomic Pathology and Pathological Sciences, Graduate School of Medical Sciences, Kyushu University, Fukuoka, JPN; 5 Department of Radiology, Faculty of Medicine, Saga University, Saga, JPN

**Keywords:** adult-type diffuse glioma, cystathionine, glioblastoma idh-wildtype, high grade glioma (hgg), magnetic resonance spectroscopy, oligodendroglioma idh-mutant and 1p/19q-codeleted

## Abstract

Oligodendroglioma, IDH-mutant and 1p/19q-codeleted, is a subtype of adult-type diffuse gliomas that demonstrates a favorable prognosis and chemosensitivity. Typical imaging findings include a location in the frontal lobe and calcification on computed tomography (CT). However, differentiating it from other gliomas, such as glioblastoma, can be difficult when characteristic imaging features, like calcification, are absent.

We present a case of a woman in her 30s with a ring-enhancing lesion in the right frontal lobe, initially suggestive of glioblastoma. Proton magnetic resonance spectroscopy (^1^H-MRS) was performed using a single-voxel, point-resolved spectroscopy sequence (PRESS) with an echo time of 97 ms. Spectral data were analyzed with LCModel (Stephen Provencher, Inc., Oakville, Canada), which revealed a distinct cystathionine peak at 2.7 ppm. This finding raised the suspicion of oligodendroglioma, which was confirmed by histopathological and molecular analyses. Cystathionine accumulation is attributed to the co-deletion of chromosome arm 1p, leading to downregulation of phosphoglycerate dehydrogenase and cystathionine gamma-lyase, and a metabolic shift toward the transsulfuration pathway. Although glioblastomas may also upregulate this pathway to resist ferroptosis, a previous study showed higher cystathionine levels in oligodendrogliomas. This case suggests that cystathionine detection by ^1^H-MRS could serve as a useful radiological clue in gliomas with atypical imaging features, which are a ring enhancement without calcification, assisting in the differential diagnosis.

## Introduction

Adult-type diffuse gliomas have three major entities: astrocytoma, IDH-mutant; oligodendroglioma, IDH-mutant and 1p/19q-codeleted; and glioblastoma, IDH-wildtype [1-3]. These tumors differ markedly in their biological behavior, prognosis, and response to chemoradiotherapy [4]. In particular, oligodendrogliomas are known to respond favorably to combined chemoradiation, making accurate histological classification critical in determining the optimal treatment strategy and surgical approach [5]. Therefore, reliable preoperative diagnosis plays a pivotal role in clinical decision-making, including determining the extent of tumor resection [6].

Proton magnetic resonance spectroscopy (^1^H-MRS) has emerged as a valuable tool in the non-invasive assessment of brain tumors [7]. Recent studies have reported that oligodendrogliomas exhibit elevated levels of cystathionine, resulting in a characteristic peak on ^1^H-MRS at 2.7 ppm, which may aid in the differentiation of adult-type diffuse gliomas [8]. However, to our knowledge, there have been no reports of the application of cystathionine peak detection in gliomas presenting with ring-enhancing lesions on conventional contrast-enhanced T1-weighted imaging - a pattern more commonly associated with high-grade gliomas, such as glioblastoma.

In this report, we present a case in which cystathionine peak detection by ^1^H-MRS contributed to the preoperative diagnosis of oligodendroglioma, despite other radiological findings suggestive of glioblastoma. This case suggests that cystathionine detection by ^1^H-MRS could serve as a useful radiological clue in gliomas with atypical imaging features - specifically, ring enhancement without calcification - assisting in the differential diagnosis.

## Case presentation

A woman in her 30s presented with neck pain, followed two months later by the onset of headache and nausea. Brain computed tomography (CT) revealed an intracranial lesion in the bilateral frontal lobes with mild edema. Her past medical history was notable only for an inguinal hernia repair two years prior, and no significant abnormalities were found in routine laboratory tests.

**Figure 1:**
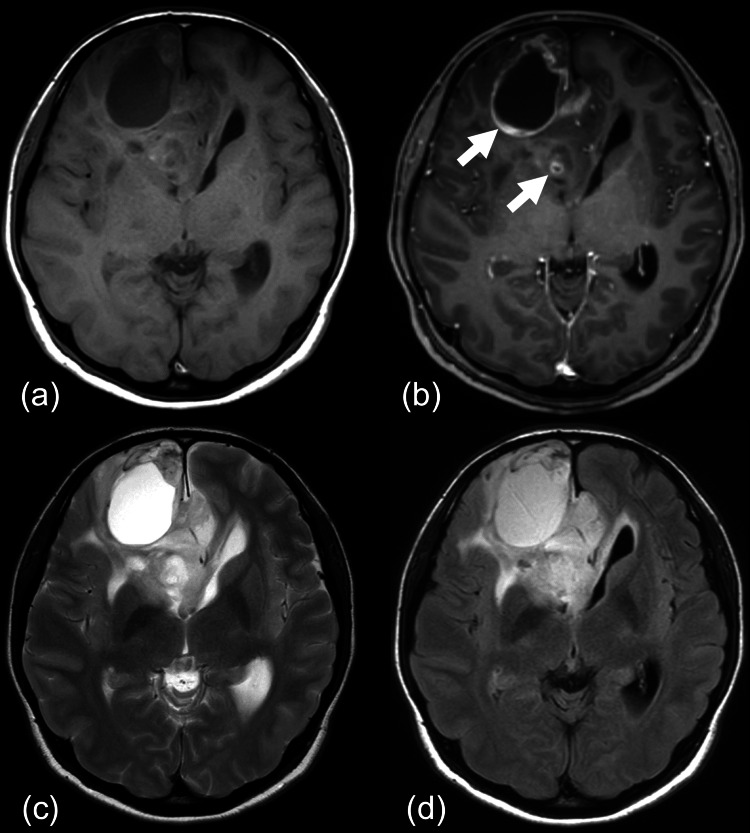
MRI scans Pre- and post-contrast T1-weighted images demonstrate irregular ring-enhancing lesions (arrows), with mass effect and involvement of the corpus callosum (a, b). MRI reveals a necrotic lesion in the right frontal lobe, with an ill-defined T2-hyperintense region (c, d). MRI, Magnetic resonance imaging

**Figure 2:**
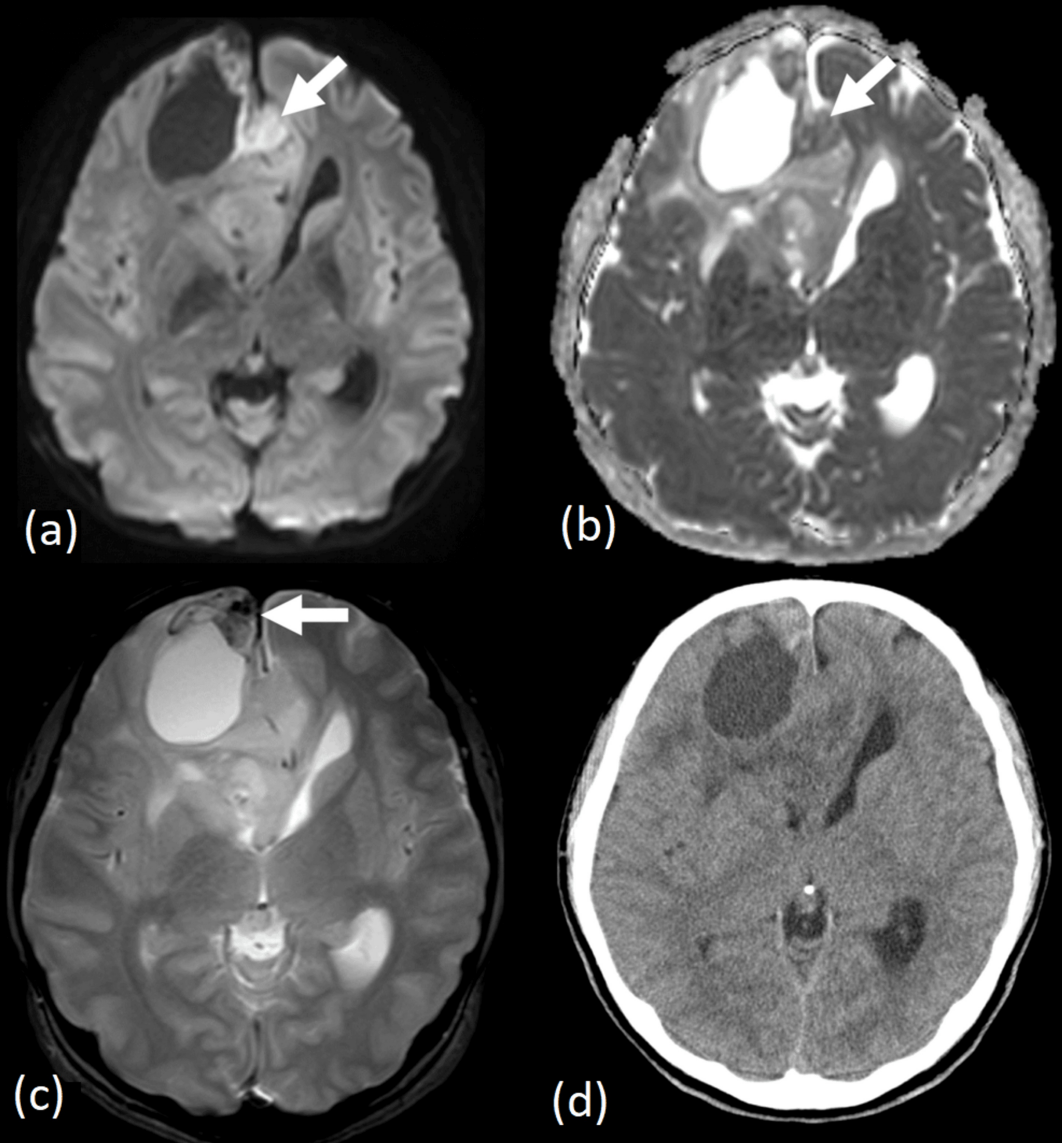
CT and MRI images Diffusion-weighted imaging shows restricted diffusion within the enhancing region (a; arrow), with a minimum apparent diffusion coefficient of 0.73 × 10⁻³ mm²/s (b; arrow). T2*-weighted imaging reveals a hypointense area in the anteromedial portion of the tumor, suggestive of intratumoral hemorrhage (c; arrow). Non-contrast CT demonstrates a 37-mm lesion, with necrotic components but no obvious calcification (d). MRI, Magnetic resonance imaging; CT, Computed tomography

**Figure 3:**
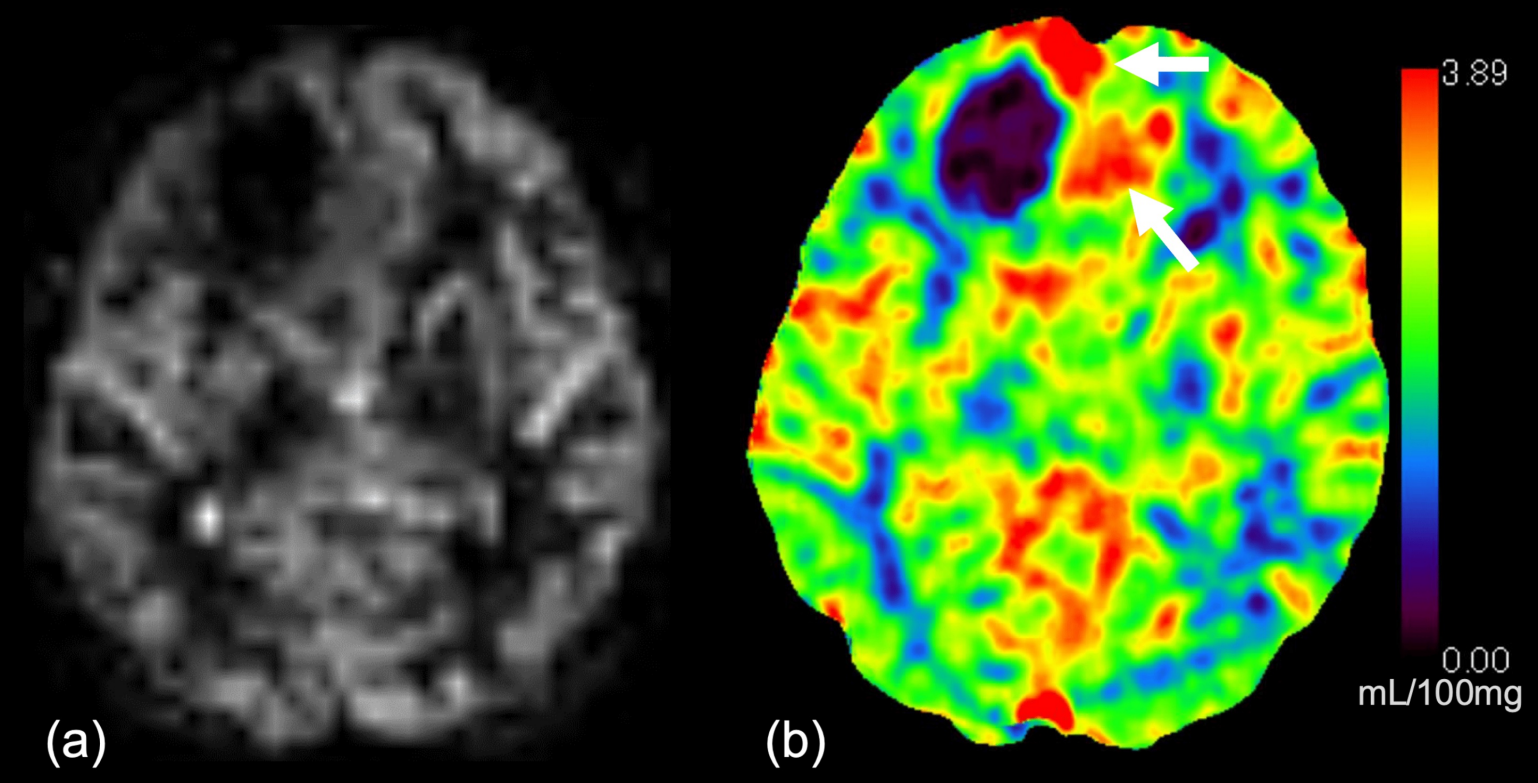
ASL and CTP images Pseudo-continuous ASL demonstrates no evidence of a cortical high-flow sign, a feature that has been reported in association with oligodendrogliomas, IDH-mutant and 1p/19q-codeleted (a). Cerebral blood volume from CTP mapping reveals increased perfusion in the medial portion of the tumor (b; arrows). ASL, Arterial spin labeling; CTP, Computed tomography perfusion

Magnetic resonance imaging (MRI; 3-tesla Ingenia Elition X 3.0 T; Philips Healthcare, Best, the Netherlands) revealed a necrotic lesion in the right frontal lobe (Figure 1), accompanied by an ill-defined T2-hyperintense area (Figures 1c-1d). Post-contrast T1-weighted imaging showed irregular, ring-enhancing lesions with associated mass effect, causing a right-to-left midline shift (Figure 1b). Diffusion-weighted imaging revealed restricted diffusion corresponding to the enhancing area (Figure 2a), with a minimum apparent diffusion coefficient (ADC) value of 0.73 × 10^-3^ mm^2^/s (Figure 2b). Gradient-echo T2-weighted imaging demonstrated a hypointense area in the anterior-medial portion of the tumor, suggesting intratumoral hemorrhage (Figure 2c). Pseudo-continuous arterial spin labeling showed no evidence of a cortical high-flow sign (Figure 3a), which suggests oligodendroglioma [9]. CT imaging showed a 37 mm mass in the right frontal cortex and subcortical white matter region, containing necrotic components but no obvious calcification (Figure 2d). The cerebral blood volume, measured by CT perfusion imaging, demonstrated increased perfusion in the medial portion of the tumor (Figure 3b).

**Figure 4:**
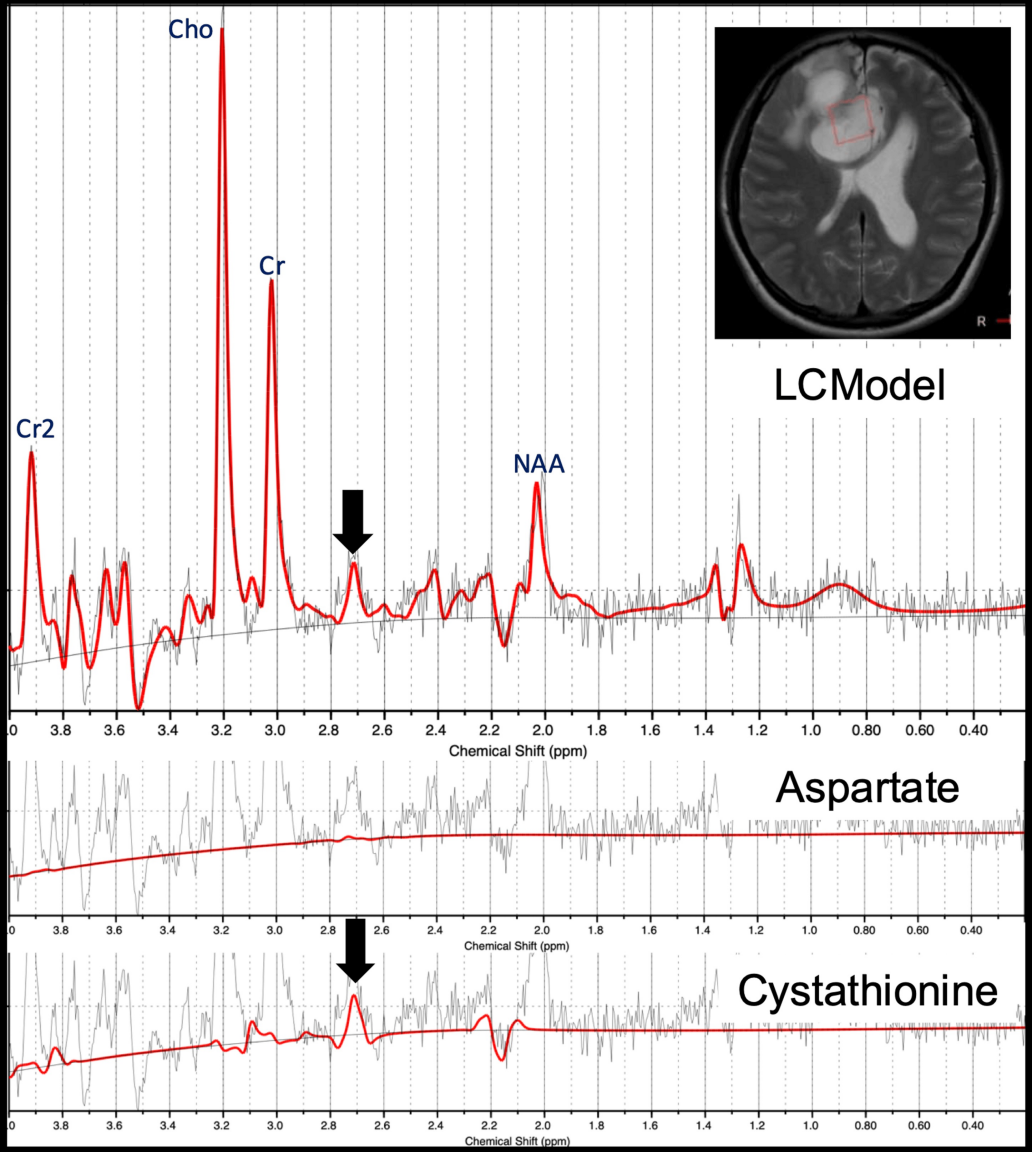
1H-Magnetic resonance spectroscopy ^1^H-magnetic resonance spectroscopy analyzed with LCModel demonstrates a distinct peak at 2.7 ppm (upper row; arrow). Fitting with aspartate does not reproduce the peak at 2.7 ppm (middle row), whereas fitting with cystathionine reveals a prominent peak at this frequency (bottom row; arrow), supporting the specificity of cystathionine accumulation in the tumor. The concentration of cystathionine is 1.6 mM in this patient.

^1^H-MRS was performed (Figure 4), and metabolic quantification was conducted using LCModel (version 6.3-1R; Stephen Provencher, Inc., Oakville, Canada) [10]. ^1^H-MR spectra were acquired using a single-voxel, point-resolved spectroscopy sequence (PRESS) (repetition time: 2000 ms; echo time: 97 ms; echo time 1: 32 ms; echo time 2: 65 ms) [11]. The total acquisition time was 4 minutes and 52 seconds. The volume of interest (VOI) was 20 × 20 × 20 mm^3^ and was placed in the intratumoral area, as referenced from conventional images. All spectra were analyzed using LCModel [10]. To estimate the concentration of cystathionine, the signal was normalized to the unsuppressed water signal, assuming a water concentration of 43.3 M in the tumor region. Relaxation corrections were applied using T_1_ and T_2_ values for both water and cystathionine, as reported in previous literature [8,12,13]. It is important to note that the estimated concentrations of cystathionine are semi-quantitative. The reliability of the cystathionine spectral fit was assessed using the Cramér-Rao lower bounds (CRLBs), expressed as the percent standard deviation (%SD) in the LCModel output file. Our study included MRS data only in cases where the %SD of cystathionine was below 50% [8]. ^1^H-MRS in this patient revealed a distinct peak at 2.7 ppm (Figure 4, upper row). Fitting with cystathionine alone (Figure 4, bottom row) revealed a prominent peak at 2.7 ppm, indicating elevated cystathionine levels. Additional fitting with aspartate did not show a peak at 2.7 ppm (Figure 4, middle row), supporting the specificity of the cystathionine elevation [8]. The concentration of cystathionine in this patient was 1.6 mM. The concentration of 2-hydroxyglutarate, a known IDH-mutation biomarker, was 0.41 mM; however, the CRLB was 149%, so this value was discarded.

Given the absence of calcification and the presence of irregular ring enhancement, glioblastoma was initially suspected. However, the detection of a cystathionine peak by ^1^H-MRS also led us to consider oligodendroglioma, IDH-mutant and 1p/19q-codeleted, in the preoperative differential diagnosis, and surgical resection was performed. Histopathological examination revealed no calcification but showed microvascular proliferation, nuclear atypia, and necrosis. Immunohistochemistry showed positive staining for IDH1 R132H, indicating an IDH-mutant tumor. Further molecular analysis resulted in the final diagnosis of oligodendroglioma, IDH-mutant and 1p/19q-codeleted, CNS WHO Grade 3.

## Discussion

Accurate preoperative diagnosis of glioma subtypes is crucial for determining the appropriate surgical approach and predicting therapeutic responses [4]. While oligodendrogliomas are known for their favorable prognosis and chemosensitivity [5], distinguishing them from other gliomas based solely on conventional imaging can be challenging. ^1^H-MRS with cystathionine quantification has emerged as a valuable imaging method. A recent study has reported that elevated cystathionine levels are highly sensitive (92%) but moderately specific (61%) for diagnosing oligodendroglioma [8], demonstrating its usefulness as a screening tool. In this case, the cystathionine concentration was 1.6 mM, which falls within the reported range for 1p/19q-codeleted gliomas (mean 2.33 ± 0.98 mM) [8]. The detection of a cystathionine peak at 2.7 ppm by ^1^H-MRS provided an important clue toward preoperative suspicion of oligodendroglioma, even though radiologic features more closely resembled those of glioblastoma. This metabolic insight facilitated a broader differential diagnosis and may have aided in a more precise preoperative diagnosis.

In this case, conventional imaging modalities lacked characteristic features typically associated with oligodendroglioma, except for age and tumor location. There was no calcification on CT, a low ADC value, and ring enhancements. Calcification - a well-recognized hallmark of oligodendroglioma - has been reported with a sensitivity of 56% in pathology [14] and up to 90% on CT [15]; however, it was not observed in this patient on either pathology or CT. Thus, ^1^H-MRS may compensate for the lack of calcification by providing a surrogate metabolic biomarker that supports the diagnosis when conventional hallmarks are missing. The 2-hydroxyglutarate, known as a biomarker for IDH mutation, was also unreliable in this case due to a high CRLB. This poor reliability was likely related to tumor heterogeneity, as necrotic and enhancing components could dilute or obscure metabolite signals. Contrast enhancement is observed in approximately half of oligodendrogliomas [16]. However, this case exhibited ring enhancement, which is rare in oligodendrogliomas (up to 20% in anaplastic cases; CNS WHO Grade 3 [16]) but frequent in glioblastomas, IDH-wildtype. This finding was supported by histopathology showing necrosis - a feature common in higher-grade gliomas [1]. The cortical high-flow sign on arterial spin labeling MRI, present in approximately 56% of 1p/19q-codeleted oligodendrogliomas [9], was also absent. Collectively, these findings initially favored a diagnosis of glioblastoma over oligodendroglioma, IDH-mutant and 1p/19q-codeleted.

In vivo ^1^H-MRS, however, revealed a distinct cystathionine peak at 2.7 ppm, a metabolic signature linked to 1p/19q-codeleted gliomas [8]. This accumulation is thought to result from co-deletion of chromosome arm 1p, leading to downregulation of phosphoglycerate dehydrogenase (1p12) and cystathionine gamma-lyase (1p31) [8], which shifts metabolism toward the trans-sulfuration pathway and elevates cystathionine levels [17]. Detection of this peak provided a crucial diagnostic clue, illustrating how ^1^H-MRS can refine the differential diagnosis in cases with atypical conventional imaging features. Therefore, while ring enhancement may raise suspicion for glioblastoma, it should not exclude the possibility of oligodendroglioma, particularly in the presence of molecular or metabolic markers suggestive of this diagnosis.

Although the detection of a cystathionine peak supported the diagnosis of oligodendroglioma in this case, it is important to acknowledge a potential limitation. Glioblastomas, IDH-wildtype, may also exhibit elevated cystathionine due to activation of the transsulfuration pathway to resist ferroptosis, with cystathionine gamma-lyase acting as a metabolic bottleneck [17]. Data on its frequency in glioblastomas, IDH-wildtype, remain limited. Branzoli et al. reported a concentration of 4.5 mM in a single glioblastoma case [18], and more recently, Chan et al. observed cystathionine in six glioblastomas, with a median (interquartile range, IQR) concentration of 1.4 (0.8-2.7) mM [19]. A previous study also demonstrated significantly higher cystathionine levels in oligodendroglioma, IDH-mutant and 1p/19q-codeleted, with superior sensitivity [20]. These findings suggest that, despite potential overlap, cystathionine quantification by ^1^H-MRS remains a useful adjunct for the non-invasive differentiation of glioma subtypes. Another limitation is the lack of comparative data or validation in larger cohorts. To date, reports on cystathionine detection in gliomas remain limited to small case series, and its diagnostic performance has not been systematically established.

## Conclusions

Although this report describes a single case, and cystathionine elevation has occasionally been observed in glioblastomas, the semi-quantitative nature of MRS measurements limits generalizability. Nevertheless, this case suggests that cystathionine detection by ^1^H-MRS could serve as a useful radiological clue in gliomas with atypical imaging features, such as ring enhancement without calcification, assisting in the differential diagnosis. Even in cases presenting with ring enhancement or lacking calcification, the combined assessment of patient age (younger), tumor location (frontal lobe), and cortical involvement - together with cystathionine detection - may improve diagnostic confidence and help distinguish oligodendroglioma from other gliomas.
